# Identifying and Categorizing Adverse Events in Trials of Digital Mental Health Interventions: Narrative Scoping Review of Trials in the International Standard Randomized Controlled Trial Number Registry

**DOI:** 10.2196/42501

**Published:** 2023-02-22

**Authors:** Aislinn D Gómez Bergin, Althea Z Valentine, Stefan Rennick-Egglestone, Mike Slade, Chris Hollis, Charlotte L Hall

**Affiliations:** 1 National Institute for Health and Care Research MindTech MedTech Co-operative Institute of Mental Health, School of Medicine University of Nottingham Nottingham United Kingdom; 2 National Institute for Health and Care Research Nottingham Biomedical Research Centre Institute of Mental Health University of Nottingham Nottingham United Kingdom; 3 Mental Health and Clinical Neurosciences School of Medicine University of Nottingham Nottingham United Kingdom; 4 School of Health Sciences Institute of Mental Health University of Nottingham Nottingham United Kingdom; 5 Faculty of Medicine and Health Sciences Nord University Namsos Norway

**Keywords:** adverse events, harm, psychological interventions, clinical trials, review, digital, mobile phone

## Abstract

**Background:**

To contextualize the benefits of an intervention, it is important that adverse events (AEs) are reported. This is potentially difficult in trials of digital mental health interventions, where delivery may be remote and the mechanisms of actions less understood.

**Objective:**

We aimed to explore the reporting of AEs in randomized controlled trials of digital mental health interventions.

**Methods:**

The International Standard Randomized Controlled Trial Number database was searched for trials registered before May 2022. Using advanced search filters, we identified 2546 trials in the category of mental and behavioral disorders. These trials were independently reviewed by 2 researchers against the eligibility criteria. Trials were included where digital mental health interventions for participants with a mental health disorder were evaluated through a completed randomized controlled trial (protocol and primary results publication published). Published protocols and primary results publications were then retrieved. Data were extracted independently by 3 researchers, with discussion to reach consensus when required.

**Results:**

Twenty-three trials met the eligibility criteria, of which 16 (69%) included a statement on AEs within a publication, but only 6 (26%) reported AEs within their primary results publication. Seriousness was referred to by 6 trials, relatedness by 4, and expectedness by 2. More interventions delivered with human support (9/11, 82%) than those with only remote or no support (6/12, 50%) included a statement on AEs, but they did not report more AEs. Several reasons for participant dropout were identified by trials that did not report AEs, of which some were identifiable or related to AEs, including serious AEs.

**Conclusions:**

There is significant variation in the reporting of AEs in trials of digital mental health interventions. This variation may reflect limited reporting processes and difficulty recognizing AEs related to digital mental health interventions. There is a need to develop guidelines specifically for these trials to improve future reporting.

## Introduction

### Background

Digital mental health interventions provide information, support, and therapy for mental health problems via a technology or a digital platform [1]. These interventions come in various formats and may be delivered via an app, smartphone, computer, or tablet. These interventions may be self-guided, with little or no human interaction, thus offering the potential to treat large numbers of patients across geographically disperse and remote locations. An automated approach can reduce staff time and costs, allowing more control over the content and delivery of a mental health intervention. However, the increasing popularity and unique delivery of digital mental health interventions pose new challenges for the monitoring and reporting of harms in clinical trials. Several of these have been highlighted in a previous consensus statement [2]. Specifically, risks may arise in the absence of staff being present to support emotional processing, explain treatment content, or guide patients in how to engage with the technology, leading to a greater risk of misunderstanding or inadequate delivery of the intervention. Given the potential differences that need to be considered when delivering mental health interventions digitally, including the potential lack of direct human contact, it may be particularly difficult for researchers to understand how best to recognize, record, and categorize adverse events (AEs) [2]. This is in addition to the existing challenges recognized by mental health researchers in AE reporting, regardless of whether the intervention is digital. Reporting of AEs in clinical trials should conform to the Consolidated Standards Of Reporting Trials (CONSORT) harms statement [3]. There is also a specific CONSORT-eHealth for reporting trials of digital health interventions, which reflects the importance of recording harms [4]. To fully report AEs, researchers must have in place appropriate definitions for how to identify and measure AEs and how to categorize them to determine seriousness, expectedness, and relatedness to the intervention. The Good Clinical Practice guidelines of the International Council for Harmonization define an AE as an “untoward medical occurrence;” a serious AE (SAE) as “death/life threatening, inpatient hospitalization, disability, birth defect,” or a “medically important event;” and “unexpected” when a reaction is not consistent with the outcomes expected [5]. However, these guidelines were developed for drug-related incidences. Applying guidance from drug trials does not always fit for mental health interventions, with risks often not relevant to the intervention, leading researchers to call for a broader and more appropriate interpretation to include areas such as additional treatment needs and life changes [6-8]. Defining what constitutes an AE and determining its relationship to the intervention usually requires a plausible sequence of events, whereby the intervention is directly linked to the AE. This is easier to define with pharmacological interventions, where the mechanisms of action are better understood [9,10].

Recent reviews of protocols for trials of mental health interventions found significant variations in how AEs were defined [7,8]. Considering the lack of consensus regarding AEs within mental health intervention research and the reliance of reporting guidelines such as CONSORT on trial teams to predefine AEs, this is concerning as it suggests the potential for underreporting. A 2014 review [11] found only 1 mental health intervention trial had been terminated on the grounds of unacceptable AEs, with very few considering harms relevant to psychological therapies such as distress or self-harm and no primary results publication reporting AEs. A review of protocols suggests an increase in the plans to record AEs [8], but it is unclear if this has translated to an increase in the actual reporting. AE reporting can be time-intensive for researchers and is arguably more straightforward when an intervention is delivered in-person, wherein AEs can be spontaneously reported or identified. However, digital mental health interventions often have reduced or no direct contact and there may be additional “unwanted events” [2] related to the digital nature of delivery (eg, technical issues). Research has previously indicated that monitoring reasons for withdrawal can highlight potential AEs, suggesting that this may be one way to identify unrecognized AEs [12]. The monitoring and reporting of AEs in trials for digital mental health interventions is a necessity to fully understand the risk-benefit ratio of such interventions. The current lack of clear guidelines for AEs precludes this from happening.

### Objectives of This Review

This scoping review aims to explore how AEs are currently assessed in trials of digital mental health interventions according to their protocols and primary results publications. Further objectives of this review were to (1) identify how AEs are reported; (2) identify how AEs are defined and classified with regard to their seriousness, expectedness, and relatedness; (3) explore how AEs are monitored; (4) explore potential harms identified within reasons given for dropout and to better understand how researchers might employ certain strategies to mitigate potential risk, for example, through excluding those most at-risk; and (5) explore whether there was a link between what research teams set out to measure in their protocol and what they reported in their final report. This review focused on trials of digital mental health interventions; therefore, it was outside the scope of this study to review variations in trials that adopt different web-based or digital methods of data collection. Furthermore, this study did not aim to compare with trials of nondigital interventions.

## Methods

### Study Design

We hosted our protocol in the public domain [13]. We made 2 deviations from this protocol; we chose to limit only to published protocols, as this best fitted with understanding reporting trial procedures, and we chose to expand our review of AEs to look at the categorization of expectedness as well as the monitoring of AEs and potential AEs to provide a more complete picture. This review was a scoping review [14] conducted in 1 database with systematic screening of records for eligibility.

### Searches

**Figure 1:**
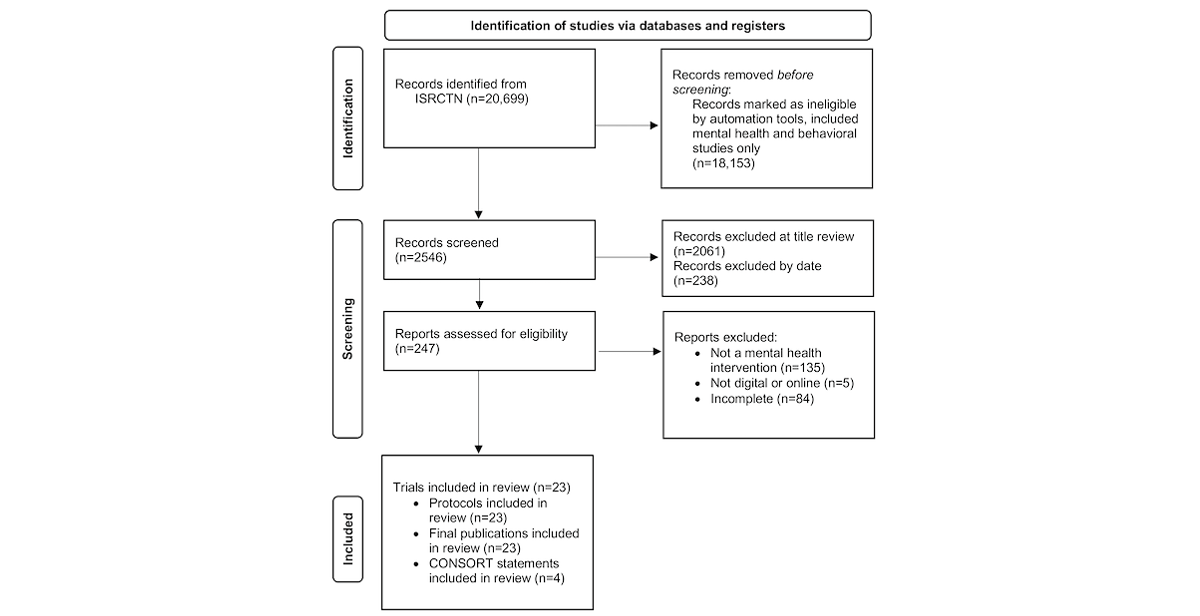
PRISMA (Preferred Reporting Items for Systematic Reviews and Meta-Analyses) diagram. CONSORT: Consolidated Standards Of Reporting Trials; ISRCTN: International Standard Randomized Controlled Trial Number.

The International Standard Randomized Controlled Trial Number (ISRCTN) registry was searched to identify relevant trials (May 2021). This registry was selected as the database to search, as it is the primary clinical trial registry recognized by the World Health Organization and the International Committee of Medical Journal Editors, and it provides a source for identifying clinical research studies across multiple sites, funders, settings, and conditions. The advanced search facility was used, wherein filters included trials only in the category of mental and behavioral disorders (see Figure 1 [15]). As such, there were no search terms, but all records that were brought up using this filter were searched.

Titles and ISRCTN abstracts were read by 2 authors (CLH and ADGB). Where it was not certain whether studies met the inclusion criteria, the plain English summary was additionally read to ascertain relevance. Reasons for exclusion were noted. A list of eligible trials was generated, and attempts were made to access (from the corresponding author and online) the published protocol and the main outcome paper(s) (ie, the paper(s) that present data from the primary outcome). The search was initially conducted on May 17, 2021, and updated on May 23, 2022, by identifying any trial that had been marked as incomplete during the previous searches and by checking for primary results publications.

### Inclusion and Exclusion Criteria

Searches were restricted from 2004 (publication year of harm-reporting extension of the CONSORT statement) to May 17, 2021. In line with previous research, exclusions were made where there are additional physical health needs that may make AE reporting more common [12]. Interventions that act as an adjunct, where most of the intervention is delivered by a clinician, were excluded. Only complete trials were included, defined as those with both a published peer-reviewed protocol and primary results publication. An eligibility check on the full protocol(s) and paper(s) was conducted by 3 researchers independently with 99.2% agreement (ADGB, AZV, CLH). Any disagreement or discrepancy was resolved through discussion by all 3 where necessary. See Table 1 for the complete list of inclusion and exclusion criteria.

**Table 1:** Inclusion and exclusion criteria.

Criteria	Inclusion criteria	Exclusion criteria
Participants, population	People (any age and any gender) who fulfilled the diagnostic criteria for a mental health disorder according to the following International Classification of Diseases, tenth revision criteria. F20-29: schizophrenia, schizotypal and delusional disorders; F30-39: mood (affective) disorders, anxiety disorders (including obsessive-compulsive disorder); F40-48: neurotic, stress-related, and somatoform disorders; F50: eating disorders; F60-62: specific personality disorders (but excluding F63-69; F63: habit and impulse disorders, F64-69: gender-related disorders); F80-89: disorders of psychological development; F90-98: behavioral and emotional disorders with onset usually occurring in childhood and adolescence.	Not mental health patient, that is, research looks at health care providers, or parents/carers. People (any age and any gender) who fulfilled the diagnostic criteria for a mental health disorder according to the following International Classification of Diseases tenth revision criteria. F00-09 relate to organic mental disorders (eg, dementia, traumatic brain injury), F10-19: mental and behavioral disorders due to psychoactive substance use, F51-59: behavioral syndromes associated with physiological disturbances and physical factors (including sleep disorders), F70-79: mental retardation, F99: unspecified mental disorder.
Interventions, exposures	Any randomized controlled trial (RCT) (including definitive, pilot, feasibility, and exploratory RCTs) of psychological interventions (ie, non-Clinical Trial of an Investigational Medicinal Product Trial, including behavioral, cognitive, and psychosocial interventions) delivered online or digitally (ie, websites, apps, synchronous and asynchronous delivery, including both with and without human support) with a published protocol and final results.	No psychological therapeutic intervention, excludes activity-based interventions, interventions focused on vocational outcomes, and interventions using herbal/alternative medicines or pharmaceutical drug trials. Also excludes preventative trials on populations at risk. Excludes assessment, adjunct use, and medication management trials, or studies. Nonrandomized trials, observational studies, and expanded access studies. Studies published prior to 2004.
Comparators, control, context	Any control group and any setting will be considered, including health services, community or school settings, prison/forensic facilities. Research in all countries will be included.	N/A^a^
Outcomes	All outcomes related to adverse events were included: reporting, definitions, seriousness, expectedness, relatedness—along with reasons for dropout.	Studies recorded as “not yet recruiting” or “ongoing.”

^a^N/A: not applicable.

### Data Extraction (Selection and Coding)

Relevant data were extracted independently by 2 researchers (ADGB and CLH) from all published trial documents, including the protocol, primary results publication, and any supplementary materials, including CONSORT checklists. Study quality was not assessed for risk of bias, as the purpose of this review was to identify information relevant to AEs rather than treatment outcomes. Categories of data for extraction are included in Multimedia Appendix 1.

## Results

### Overview

Our search is reported according to the PRISMA (Preferred Reporting Items for Systematic Reviews and Meta-Analyses) checklist [15]. It identified 2546 unique citations (see Figure 1). We excluded 238 that did not meet the date criteria, 2061 after screening titles, and 224 after screening full text because they did not meet the inclusion criteria. Finally, 23 trials were identified (see Multimedia Appendix 2 for references) [16-38]. Their characteristics, including details on participant condition and intervention, are shown in Multimedia Appendix 3. Most interventions were delivered using the web/internet via either an app or through a web browser on a computer, laptop, tablet, or smartphone [17-24,26-34,36-38]. Three were delivered using virtual reality equipment [16,25,35]. Sixteen trials recruited adults older than 18 years [16-22,24,25,27-29,33,34,36,37], with a further 4 trials including those older than 16 years [26,31,35,38], and 3 trials with those younger than 18 years only [23,30,32].

### Studies With AEs

Multimedia Appendix 4 outlines how AEs were reported, defined, monitored, and how seriousness, expectedness, and relatedness were addressed in the trials. Of the 23 trials, 7 did not mention AEs within any trial documentation [17-19,22,25,34,36]. Of the remaining 16 trials, 10 mentioned AEs in both the trial protocol and primary results publication [16,21,27-31,33,35,38], with 2 additionally mentioning AEs within a CONSORT checklist [31] and in the ISRCTN database [30]. One trial only referred to AEs within their primary results publication [32], 1 only within their protocol [23], and 3 only within a supplementary CONSORT checklist provided alongside their primary results publication [20,24,37]. The remaining trial mentioned AEs within their protocol and a CONSORT checklist [26]. Seven studies that included younger adults and children all mentioned AEs [23,26,30-32,35,38].

### Reporting of AEs

Fifteen trials reported on the presence/absence of AEs within their primary results publication [16,20,21,24,26-33,35,37,38]. Seven of these trials reported no AEs [16,24,26,31-33,38]. Two trials reported that AEs were not applicable [20,37]. Four trials reported both AEs and SAEs [21,27,30,35] and 2 trials only reported AEs [28,29].

### How AEs Were Defined

A definition of what classified as an AE was provided within the protocol of 8 trials, with 1 further trial doing so within their primary results publication [21,23,27-31,33,35]. However, there was a considerable variation. Three trials used a symptom checklist to define an AE, one of which did not then report AEs within their primary results publication [23,29,30]. Five trials identified fatal or life-threatening events but differed in the additional definitions [21,23,27,31,35]. One identified only in their primary results publication how AEs were defined, as deterioration in the primary outcome measure [28]. Two trials directly considered AEs specific to the device [31,32].

### Seriousness of AEs

Six of the 14 trials that mentioned AEs referred to the seriousness of AEs, although one did not provide information as to how seriousness would be determined—only that it would be rated by severity [21,29-31,35,38]. Five provided a definition of seriousness, and of these, 4 stated that the event would be reviewed by a senior member of the research team. SAEs were defined as fatal (n=4), life-threatening (n=4), hospitalization (n=4), disability (n=3), congenital (n=2), and an event identified by a senior member of the trial team as serious (n=3).

### Expectedness

One trial reported expected and unexpected AEs separately in their primary results publication [30]. An additional trial mentioned in their protocol that the investigators would determine if an AE was expected or unexplained; however, these were not reported in the primary results publication [27]. Although not explicitly referring to categorizing the event as it occurs, a further trial stated they would only monitor AEs inherent to the nature of the condition, which implies some form of expectedness [23]. One further trial stated they did not expect AEs [33].

### Monitoring of AEs

Eight trials provided information on the monitoring and, in some cases, reviewing of AEs [21,23,27,29-31,35,38]. In 1 trial, AEs were monitored by a mental health worker in each session and throughout by reporting to a therapist, outcome assessor, or by self-report. One trial reviewed medical notes at the end of the trial [35]. Three used checklists [23,29,30], including the Short Mood and Feelings Questionnaire in every session [39], Generalized Anxiety Disorder [40] and Patient Health Questionnaire [41] that were provided frequently, a modified symptom checklist postintervention [42], and a modified side-effects scale used throughout and for 3 months after the intervention finished [43]. Potential AE/SAEs were also reported to and reviewed by senior team members (eg, the chief investigator) or external committees (n=8) [21,23,27,29-31,35,38]. One indicated that they were monitoring AEs using existing guidance [27] (Standard Protocol Items: Recommendations for Interventional Trials) [44]. One study indicated that it would be monitored through self-report [33].

### Relatedness of AEs

Five trials addressed how related an event was to the intervention or trial procedures [27,29-31,35]. The process for identifying relatedness involved an assessment of the event by the research team in consideration of the participant’s individual situation (eg, their condition, treatment), the temporal relatedness to the trial, discussion with parents/carers/participants, and an assessment by independent committees or trial investigators.

### Potential AEs

To help us identify whether harms that may lead to AEs are not being recognized, recorded, or reported, we further scrutinized the data to highlight those areas identified in previous research as potentially related to AEs. This information is summarized in Multimedia Appendix 5 (studies that did not reference or report AEs, n=17) and Multimedia Appendix 6 (studies that reported AEs, n=6), in terms of support and training, dropouts, strategies to manage risk and eligibility criteria, alongside whether the trial identified any AEs.

### Support

Providing support alongside an intervention offers more opportunities for contact with participants, potentially increasing the reporting of AEs. Direct support was defined as support provided in-person, for example, with someone delivering the intervention or existing services providing assistance. Brief support included support provided in-person but only to demonstrate or introduce the intervention. Remote support included delivery of support via digital methods, for example, by email. Eleven trials provided remote support [18-20,22,26,28,30,33,34,36,37], 7 trials provided direct support [16,23,25,27,31,32,35], and 4 trials provided brief support [17,21,24,38]. One did not provide support [29]. Trials that offered direct (6/7, 86%) and brief support (3/4, 75%) mentioned AEs more than those with remote support (5/11, 45%) although they did not report experiencing more AEs within the primary results publication. The trial that did not include support mentioned AEs [29].

### Dropouts and Noncompleters

Five trials did not report on reasons for dropout and did not mention AEs [17-19,22,36]. One trial that stated there had been no AEs within the study [24] and another that indicated that AEs were not applicable [37] did not report on dropouts. Ten trials reported on reasons for dropout and did not mention or report AEs [16,20,23,25,26,31-34,38]. One trial reported on both AEs and dropouts [28]. Reasons for dropout are listed in Multimedia Appendices 5 and 6 with several identifiable as an AE/SAE within the definitions identified in our sample. Two trials that did not mention AEs reported significantly higher dropouts from the intervention arm versus those from a waitlist control [45,46]. Two that did mention AEs but stated that they were not applicable also had higher dropouts from the intervention arm compared to that from the website that was offered as a control [47,48]. One that also stated there were no AEs had a similar pattern of high dropouts in the intervention arm compared to that in the waitlist control [49].

### Risk Mitigation Strategies

We looked at possible strategies that may have been used by studies not reporting AEs to manage risks in patients during the delivery of the intervention. Possible strategies identified included reviewing all communication [18], preventing participants discussing certain topics (eg, suicide) [20], a user contract and system for flagging those considered higher need [22], asking clinicians to monitor patients [24], visible signposting within the intervention for support [34], and a panic button while in virtual reality [25]. Two virtual reality trials also included a cybersickness measure not used for AE reporting [16,25]. Eight trials excluded individuals with physical conditions that could impact their use of technologies (eg, visual problems) [16,21,24,25,27,29,32,35] or those with limited access/experience in technologies (n=9) [17, 18, 26, 28, 30, 31, 33, 34, 36]. Other common exclusion criteria included more serious and enduring illness (n=13) [18-25, 27-30, 35], substance misuse (n=7) [18, 21, 27, 28, 30, 35, 37], and suicidality (n=10) [18,19,21,22,24,28,30,35-37]. Thirteen trials screened participants for their language ability [16,17,24-26,30-32,34-38].

## Discussion

To understand the reporting of AEs in trials of digital mental health interventions, we reviewed protocols and primary results publications of the completed trials listed in the ISRCTN registry, an international clinical trial database. Our findings indicated that although two-thirds of the trials mentioned AEs, only 26% (6/23) reported experiencing an AE or an SAE in their primary results publication. Additionally, there was a lack of reporting on the categorization of the seriousness, expectedness, and relatedness of AEs. This review has highlighted several challenges associated with recognizing and monitoring AEs in trials of digital mental health interventions.

Our findings indicate new challenges in monitoring AEs in trials of digital mental health interventions. How these interventions are delivered (eg, with no direct human contact) could lead to fewer AEs being identified within trials [2], but our findings suggested that although trials of interventions that offer direct or brief support are more likely to mention AEs in their protocol, they are not more likely to record their presence in the primary results publication. The aim of this study was to review the reporting of AEs within trials of digital mental health interventions; however, it also highlights the challenges within digital trial processes. Rather than the delivery of the intervention, it may be that certain aspects of the trial (eg, online-only data collection) make it particularly difficult to assess or record negative effects and thus lead to fewer recorded AEs. However, this was outside the scope of this review.

Our findings seemed to suggest that some populations are more likely to be monitored for AEs (eg, when they are younger). We are mindful of the additional burden that monitoring AEs can place on both participants and trial teams, but this must be carefully balanced with the need for methodological improvements. Our review suggested that the functionality of digital interventions could be utilized to support more standardized methods of AE reporting and recording through automating and streamlining the reporting of AEs for participants. Automated processes could be used to collect data on harms or flag for risk of harm (eg, if participant scores meet a predefined cutoff). The development of relevant symptom checklists deliverable in a web-based format would be an important step within this field. Some work has already started on the specific checklists for psychological interventions [50]; however, to the best of our knowledge, this has not been done digitally or online.

Alongside improved monitoring, it is also important to ensure that AEs within trials of digital mental health interventions are recognized and classified appropriately [2,4,8]. Our findings indicate difficulties in classifying AEs in digital mental health, which are likely to reflect the lack of relevant guidelines. As with other reviews, we identified several reasons for dropout indicative of AEs or SAEs, but these were not categorized as such by the studies. Additionally, several studies had higher dropouts associated with the digital intervention [45-49], indicating that identifying reasons for dropout may highlight unseen or unrecognized AEs or SAEs. Indeed, a trial excluded from this review due to a focus on relapse prevention found that 2 of those who withdrew had experienced an app-related AE [51]. This study also demonstrated the potential significance of AEs related to the technology itself, with 13 AEs reported as app-related, one of which was classified as serious. Most use the standard definition from the Good Clinical Practice guidelines of the International Council for Harmonization, and our findings suggest that important AEs linked to the technology may not be recognized, for example, cybersickness. Difficulties with, restricted, or no access to a digital intervention during a trial may mean that individuals experience heightened distress, misunderstand or misuse the intervention, or feel excluded. One paper in our review highlighted this as a potential harm where the automated eligibility check excluded those with more severe depression and contributed to feelings of “disappointment, frustration and a sense of exclusion” [52]. It is possible that researchers employ certain strategies to mitigate potential harms. Some examples from this review relate to the digital nature of the intervention, for example, excluding those with limited technical ability, while others relate to mental health, for example, providing human contact or support through which risk can be assessed. Research conducted by Papaioannou and colleagues [8] suggests that feelings of failure if the mental health intervention is not successful may be an unintended consequence definable as an AE within psychological therapies. Other researchers have called for a broader definition that includes, for example, deterioration of symptoms [7] and noted several potential AEs directly related to the remote delivery of interventions, including misuse [2].

Only 4 trials referred to how related an AE was to the intervention. The causal pathways in psychological interventions are less clear-cut than those in pharmacological interventions. A “dark logic” model has previously been proposed, whereby researchers set out a priori an intervention’s mechanism of action, which can be examined for where potential harms may occur [53]. The use of Trial Steering Committees to determine relatedness can provide some independence in this process. Our review suggests that AEs may not be that common (as indicated by only 6 of the 23 trials reporting AEs) and could be reviewed by a committee. However, it is perhaps more efficient to focus on SAEs or predefine events to be reviewed and to establish a blinded end point review committee to evaluate any bias in the categorization of relatedness. Furthermore, from our own experiences conducting trials, reviewing temporal relationships between the intervention and the onset of the AE, whether the AE symptom had ever occurred before participation and asking the opinion of the participant were important in determining relatedness. However, these necessitate that direct contact is possible with participants. This can be done through the support provided, through the intervention itself, or embedded within trial processes.

Although preventative trials were outside the eligibility criteria of this review, it is likely by their very nature, they may have superior AE monitoring (ie, their primary aim is likely to be related to AEs) and thus, they may provide an opportunity for shared learning. For example, a feasibility randomized controlled trial that investigated a medical device (EMPOWER) to prevent relapse in patients with schizophrenia was particularly impressive in the AE monitoring [51]. EMPOWER included a smartphone for participants to actively monitor their symptoms, combined with peer support and clinical triage where needed. AEs were identified via contact with an unblinded peer support worker who monitored the participants during the trial via fortnightly telephone contact, and AEs were categorized according to their severity, intensity, and relatedness and included those that were app-related. By monitoring AEs routinely, the team was able to respond to the service users’ arising needs. That study illustrates the potential advantage of blended (human/digital) interventions to facilitate AE reporting. However, for resource reasons or due to the nature of the intervention, this may not always be possible. It also may be particularly challenging to do blinded, when researchers are unaware of which treatment is being received. Of interest, this intervention was also classified as a medical device, and as such, adequate AE or SAE reporting is mandated in order to satisfy regulatory approval. Two included trials are also known to us to have interventions classified as medical devices, both of which demonstrated significantly more thorough AE monitoring and reporting [27,35]. It is possible that as guidelines on what classifies as a medical device (including software as a medical device) continue to evolve, there will be greater emphasis and requirement for more research teams to be appropriately monitoring AEs or SAEs.

Unlike other studies, we believe our research is strengthened by the inclusion of both protocols and primary results publications and is the first to explore AE reporting in trials of digital mental health interventions. This allowed us to compare between what research teams set out to measure and what they reported in the final papers. However, there would be merit in a future review including all studies, regardless of whether there was an available protocol and primary results publication. A limitation may also be that our search was constrained only to trials registered on ISRCTN with a published protocol and primary results publication, excluding other registries such as ClinicalTrials.gov. It is likely that registered trials with a published protocol are conducted with higher rigor than nonregistered trials; so, our findings may overrepresent the recording of AEs. Future reviews could explore if higher quality trials were more likely to record AEs. We did not explore whether teams had recorded processes related to AEs in additional standard operating procedures, as CONSORT defines these processes should be reported with the trial findings. It was also outside of the scope of this review to explore potential AEs in detail, but the review did highlight several areas unique to digital mental health interventions that merit further research to better understand their potential for harm. We identified that human support did not appear to lead to the reporting of more AEs; however, further research should consider comparing both web-based and offline trial processes, as more contact with researchers may lead to more opportunities for identifying AEs. Further research could also explore the nature of AEs in psychological trials, which was not the aim of this review. Additionally, it would be useful to directly compare AE reporting in studies conducted online and offline.

Following Papaioannou and colleagues’ [8] recommendations for psychological trials, we provide further suggestions for AE reporting in trials of digital mental health interventions. These should support the development of specific guidance that captures their unique nature in relation to the processes of remote data collection and technological delivery.

1. Improving monitoring: It is essential that AEs are systematically monitored in trials for digital mental health interventions in a meaningful way, especially where the interventions will subsequently be offered more widely. Monitoring AEs more closely will enable researchers to identify negative effects that may not be clear otherwise.
2. Improving reporting: Trial teams need to balance the additional patient and staff burden resulting from monitoring AEs with the need to ensure accurate reporting to ensure the validity of the trial to assess the efficacy and safety of the intervention. For instance, digital mental health interventions that are delivered remotely could have clear reporting options embedded (eg, a link within a website or email) to monitor for AEs. More systematic processes unique to digital technologies could be utilized, for example, through the automation of reaching a predefined score on a risk item (ie, suicidal ideation) or an outcome measure. We endorse the need for patient and public involvement to improve understanding the burden from the patient perspective.
3. Improving classification: Mechanisms of action of the intervention should as far as possible be defined a priori to understand potential causality/relatedness of AEs, as well as exploring temporal relationships to engagement in the intervention and prior presence of the event before participation. A logic model to describe the mechanism of action could be developed upfront and explored further during the trial through a process evaluation. A process evaluation is a mixed methods approach to understand the quality of the implementation of a complex intervention as well as to understand the dose and reach of the intervention, analyze causal mechanisms, and identify contextual factors [54]. They can be used to support the interpretation of trial findings.
4. Independent review: Experts within digital mental health or at least the health-related factors associated with a technology could be consulted to support AE classification and included in independent oversight committees. This will help to predefine or identify SAEs that are unique or related to the digital mental health intervention. These should consider the type of technology.
5. Understanding dropout: Future research and implementation can be supported through the recording and reviewing of reasons for dropout to better understand the seriousness/severity from the perspective of participants and any potential AEs unique to the technology.
6. Anonymized data sharing: Anonymized data sharing initiatives may also help inform research teams on the types of AEs likely to occur in digital mental health trials.

In summary, although most published protocols of studies are giving recognition to the identification of AEs in trials of digital mental health interventions, there appears to be a gap in the reporting of AEs in their primary results publications, with even fewer studies reporting on the categorization of AEs in relation to seriousness, relatedness, or expectedness. This highlights a potential lack of knowledge in identifying and classifying AEs in digital mental health. This gap also suggests potential methodological difficulties with gathering AE data, which may be more complex in remotely conducted studies, although this is yet to be addressed within a review. There is a need to develop guidelines for AE reporting specifically for these trial designs to improve reporting practices, conform to CONSORT guidelines, and fully understand the cost-benefit of digital mental interventions.

## Appendix

Multimedia Appendix 1 Data extraction strategies.

Multimedia Appendix 2 References for the reviewed trials.

Multimedia Appendix 3 Trial characteristics.

Multimedia Appendix 4 Adverse event reporting in trials.

Multimedia Appendix 5 Potential adverse events identified in trials that made no reference to adverse events or reported no adverse events.

Multimedia Appendix 6 Potential adverse events identified in trials that reported adverse events and serious adverse events.

## Abbreviations

AE: adverse event
CONSORT: Consolidated Standards Of Reporting Trials
ISRCTN: International Standard Randomized Controlled Trial Number
PRISMA: Preferred Reporting Items for Systematic Reviews and Meta-Analyses
SAE: serious adverse event

## Data Availability

All relevant data are included in this publication. Trials included in this review are identified in the tables and multimedia appendices. All excluded studies are available on request to the corresponding author. Researchers who require further details may contact the corresponding author with a methodologically sound proposal.
