# Supplementary material for: Identifying and Categorizing Adverse Events in Trials of Digital Mental Health Interventions: Narrative Scoping Review of Trials in the International Standard Randomized Controlled Trial Number Registry
Source: JMIR Ment Health. 2023 Feb 22;10:e42501. doi: 10.2196/42501 (PMC9996423; doi:10.2196/42501)
Supplement: Multimedia Appendix 1 [file mental_v10i1e42501_app1.pdf]

Multimedia Appendix 1: Data extraction categories

An Excel database was created which included the following:

- Trial information: title, country, primary study design, aim of trial, year of trial result publication, description of comparators/control, setting.
- Intervention information: type of intervention, description of intervention, method of delivery, length and frequency of use.
- Participant information: participants, condition, method of recruitment.
- Adverse Events: where AEs were included, how AEs were defined, how AEs were identified, how seriousness was defined and reported, how relatedness was defined and reported.

  Additional data was extracted to review for potential harms.

- Support, training provided for support, contact;
- Dropouts, non-completers, withdrawals;
- Patient characteristics that may impact their use of digital technologies including access, experience, language, exclusion criteria and comorbid physical conditions.
