# Supplementary material for: Identifying and Categorizing Adverse Events in Trials of Digital Mental Health Interventions: Narrative Scoping Review of Trials in the International Standard Randomized Controlled Trial Number Registry
Source: JMIR Ment Health. 2023 Feb 22;10:e42501. doi: 10.2196/42501 (PMC9996423; doi:10.2196/42501)
Supplement: Multimedia Appendix 2 [file mental_v10i1e42501_app2.pdf]

*Multimedia Appendix 2: References for reviewed trials*

| Ref | ISRCTN Reference | Protocol Reference | Final Publication Reference |
|---|---|---|---|
| 16 | ISRCTN12929657 | Pot-Kolder, R., Veling, W., Geraets, C., & van der Gaag, M. (2016). Effect of virtual reality exposure therapy on social participation in people with a psychotic disorder (VRETp): study protocol for a randomized controlled trial. *Trials*, *17*(1), 1-9. | Pot-Kolder, R. M., Geraets, C. N., Veling, W., van Beilen, M., Staring, A. B., Gijsman, H. J., … & van der Gaag, M. (2018). Virtual-reality-based cognitive behavioural therapy versus waiting list control for paranoid ideation and social avoidance in patients with psychotic disorders: a single-blind randomised controlled trial. *The Lancet Psychiatry*, *5*(3), 217-226. |
| 17 | ISRCTN81375447 | Simpson, S., Barnes, E., Griffiths, E., Hood, K., Cohen, D., Craddock, N., … & Smith, D. J. (2009). The Bipolar Interactive Psychoeducation (BIPED) study: trial design and protocol. *BMC psychiatry*, *9*(1), 1-8. | Smith, D. J., Griffiths, E., Poole, R., Di Florio, A., Barnes, E., Kelly, M. J., … & Simpson, S. (2011). Beating Bipolar: exploratory trial of a novel internet-based psychoeducational treatment for |

| | | | |
|---|---|---|---|
| | | | bipolar disorder. *Bipolar disorders*, *13*(5-6), 571-577. |
| 18 | ISRCTN03704676 | Richards, D., Timulak, L., Doherty, G., Sharry, J., Colla, A., Joyce, C., & Hayes, C. (2014). Internet-delivered treatment: its potential as a low-intensity community intervention for adults with symptoms of depression: protocol for a randomized controlled trial. *BMC psychiatry*, *14*(1), 1-11. | Richards, D., Timulak, L., O'Brien, E., Hayes, C., Vigano, N., Sharry, J., & Doherty, G. (2015). A randomized controlled trial of an internet-delivered treatment: its potential as a low-intensity community intervention for adults with symptoms of depression. *Behaviour research and therapy*, *75*, 20-31. |
| 19 | ISRCTN24874457 | van Bastelaar, K. M., Pouwer, F., Cuijpers, P., Twisk, J. W., & Snoek, F. J. (2008). Web-based cognitive behavioural therapy (W-CBT) for diabetes patients with co-morbid depression: design of a randomised controlled trial. *BMC psychiatry*, *8*(1), 1-7. | Van Bastelaar, K. M., Pouwer, F., Cuijpers, P., Riper, H., & Snoek, F. J. (2011). Web-based depression treatment for type 1 and type 2 diabetic patients: a randomized, controlled trial. *Diabetes care*, *34*(2), 320-325. |

| | | | |
|---|---|---|---|
| 20 | ISRCTN65657330 | Griffiths, K. M., Crisp, D., Christensen, H., Mackinnon, A. J., & Bennett, K. (2010). Study protocol The ANU WellBeing study: a protocol for a quasi-factorial randomised controlled trial of the effectiveness of an Internet support group and an automated Internet intervention for depression. *BMC Psychiatry*, *10*. | Griffiths, K. M., Mackinnon, A. J., Crisp, D. A., Christensen, H., Bennett, K., & Farrer, L. (2012). The effectiveness of an online support group for members of the community with depression: a randomised controlled trial. *PloS one*, *7*(12), e53244. |
| 21 | ISRCTN40484777 | de Zwaan, M., Herpertz, S., Zipfel, S., Tuschen-Caffier, B., Friederich, H. C., Schmidt, F., … & Hilbert, A. (2012). INTERBED: internet-based guided self-help for overweight and obese patients with full or subsyndromal binge eating disorder. A multicenter randomized controlled trial. *Trials*, *13*(1), 1-13. | De Zwaan, M., Herpertz, S., Zipfel, S., Svaldi, J., Friederich, H. C., Schmidt, F., … & Hilbert, A. (2017). Effect of internet-based guided self-help vs individual face-to-face treatment on full or subsyndromal binge eating disorder in overweight or obese patients: the INTERBED randomized clinical trial. *JAMA psychiatry*, *74*(10), 987-995. |
| 22 | ISRCTN16303842 | Richards, D., Timulak, L., Doherty, G., Sharry, J., McLoughlin, O., Rashleigh, C., … & Joyce, C. | Richards, D., Timulak, L., Rashleigh, C., McLoughlin, O., Colla, A., Joyce, C., … & |

| | | | |
|---|---|---|---|
| | | (2014). Low-intensity internet-delivered treatment for generalized anxiety symptoms in routine care: protocol for a randomized controlled trial. *Trials*, *15*(1), 1-11. | Anderson-Gibbons, M. (2016). Effectiveness of an internet-delivered intervention for generalized anxiety disorder in routine care: a randomised controlled trial in a student population. *Internet interventions*, *6*, 80-88. |
| 23 | ISRCTN31219579 | Wright, B., Tindall, L., Littlewood, E., Adamson, J., Allgar, V., Bennett, S., … & Ali, S. (2014). Computerised cognitive behaviour therapy for depression in adolescents: study protocol for a feasibility randomised controlled trial. *BMJ open*, *4*(10), e006488. | Wright, B., Tindall, L., Littlewood, E., Allgar, V., Abeles, P., Trépel, D., & Ali, S. (2017). Computerised cognitive–behavioural therapy for depression in adolescents: feasibility results and 4-month outcomes of a UK randomised controlled trial. *BMJ open*, *7*(1), e012834. |
| 24 | ISRCTN82388279 | Castro, A., García-Palacios, A., García-Campayo, J., Mayoral, F., Botella, C., García-Herrera, J. M., … & Gili, M. (2015). Efficacy of low-intensity psychological intervention applied by ICTs for the | Gili, M., Castro, A., García-Palacios, A., Garcia-Campayo, J., Mayoral-Cleries, F., Botella, C., … & Baños, R. M. (2020). Efficacy of three low-intensity, internet-based psychological interventions for the treatment of depression in primary care: |

| | | | |
|---|---|---|---|
| | | treatment of depression in primary care: a controlled trial. *BMC psychiatry*, *15*(1), 1-10. | randomized controlled trial. *Journal of medical Internet research*, *22*(6), e15845. |
| 25 | ISRCTN25824611 | Raghav, K., Van Wijk, A. J., Abdullah, F., Islam, M., Bernatchez, M., & De Jongh, A. (2016). Efficacy of virtual reality exposure therapy for treatment of dental phobia: a randomized control trial. *BMC oral health*, *16*(1), 1-11. | Gujjar, K. R., van Wijk, A., Kumar, R., & de Jongh, A. (2019). Efficacy of virtual reality exposure therapy for the treatment of dental phobia in adults: A randomized controlled trial. *Journal of anxiety disorders*, *62*, 100-108. |
| 26 | ISRCTN12673428 | Kaylor-Hughes, C. J., Rawsthorne, M., Coulson, N. S., Simpson, S., Simons, L., Guo, B., … & Morriss, R. K. (2017). Direct to public peer support and e-therapy program versus information to aid self-management of depression and anxiety: protocol for a randomized controlled trial. *JMIR research protocols*, *6*(12), e8061. | Morriss, R., Kaylor-Hughes, C., Rawsthorne, M., Coulson, N., Simpson, S., Guo, B., … & Williams, L. (2021). A direct-to-public peer support program (Big White Wall) versus web-based information to aid the self-management of depression and anxiety: Results and challenges of an automated randomized controlled trial. *Journal of medical Internet research*, *23*(4), e23487. |

| | | | |
|---|---|---|---|
| 27 | ISRCTN32448671 | Garety, P. A., Ward, T., Freeman, D., Fowler, D., Emsley, R., Dunn, G., … & Hardy, A. (2017). SlowMo, a digital therapy targeting reasoning in paranoia, versus treatment as usual in the treatment of people who fear harm from others: study protocol for a randomised controlled trial. *Trials*, *18*(1), 1-13. | Garety, P., Ward, T., Emsley, R., Greenwood, K., Freeman, D., Fowler, D., … & Hardy, A. (2021). Effects of SlowMo, a blended digital therapy targeting reasoning, on paranoia among people with psychosis: a randomized clinical trial. *JAMA psychiatry*, *78*(7), 714-725. |
| 28 | ISRCTN91967124 | Richards, D., Duffy, D., Blackburn, B., Earley, C., Enrique, A., Palacios, J., … & Timulak, L. (2018). Digital IAPT: the effectiveness & cost-effectiveness of internet-delivered interventions for depression and anxiety disorders in the Improving Access to Psychological Therapies programme: study protocol for a randomised control trial. *BMC psychiatry*, *18*(1), 1-13. | Richards, D., Enrique, A., Eilert, N., Franklin, M., Palacios, J., Duffy, D., … & Timulak, L. (2020). A pragmatic randomized waitlist-controlled effectiveness and cost-effectiveness trial of digital interventions for depression and anxiety. *NPJ digital medicine*, *3*(1), 1-10. |
| 29 | ISRCTN12765810 | Gu, J., Miller, C. B., Henry, A. L., Espie, C. A., Davis, M. L., Stott, R., … & Carl, J. R. (2020). | Carl, J. R., Miller, C. B., Henry, A. L., Davis, M. L., Stott, R., Smits, J. A., … & Espie, C. A. (2020). |

| | | | |
|---|---|---|---|
| | | Efficacy of digital cognitive behavioural therapy for symptoms of generalised anxiety disorder: a study protocol for a randomised controlled trial. *Trials*, *21*(1), 1-11. | Efficacy of digital cognitive behavioral therapy for moderate-to-severe symptoms of generalized anxiety disorder: A randomized controlled trial. *Depression and anxiety*, *37*(12), 1168-1178. |
| 30 | ISRCTN70758207 | Hall, C. L., Davies, E. B., Andrén, P., Murphy, T., Bennett, S., Brown, B. J., … & Hollis, C. (2019). Investigating a therapist-guided, parent-assisted remote digital behavioural intervention for tics in children and adolescents—'Online Remote Behavioural Intervention for Tics'(ORBIT) trial: protocol of an internal pilot study and single-blind randomised controlled trial. *BMJ open*, *9*(1), e027583. | Hollis, C., Hall, C. L., Jones, R., Marston, L., Le Novere, M., Hunter, R. M., … & Murray, E. (2021). Therapist-supported online remote behavioural intervention for tics (ORBIT) in children and adolescents: A single-blind randomised controlled trial. *The Lancet Psychiatry* |
| 31 | ISRCTN10004994 | Steare, T., O'Hanlon, P., Eskinazi, M., Osborn, D., Lloyd-Evans, B., Jones, R., … & Johnson, S. (2019). App to support Recovery in Early | Steare, T., O'Hanlon, P., Eskinazi, M., Osborn, D., Lloyd-Evans, B., Jones, R., … & Johnson, S. (2020). Smartphone-delivered self-management for |

| | | | |
|---|---|---|---|
| | | Intervention Services (ARIES) study: protocol of a feasibility randomised controlled trial of a self-management Smartphone application for psychosis. *BMJ open*, *9*(3), e025823. | first-episode psychosis: the ARIES feasibility randomised controlled trial. *BMJ open*, *10*(8), e034927. |
| 32 | ISRCTN14818949 | Murphy, S., Joffe, V., Messer, D., Crafter, S., Radley, J., Sunthararajah, S., … & Welch, C. (2019). Evaluating 'enhancing pragmatic language skills for young children with social communication impairments'(E-PLAYS): protocol for a feasibility randomised controlled trial study. *Pilot and Feasibility Studies*, *5*(1), 1-13. | Murphy, S., Joffe, V., Donald, L., Radley, J., Sunthararajah, S., Welch, C., … & Torgerson, D. (2021). Evaluating 'Enhancing Pragmatic Language skills for Young children with Social communication impairments'(E-PLAYS): a feasibility cluster-randomised controlled trial. Pilot and Feasibility Studies, 7(1), 1-18. |
| 33 | ISRCTN15819951 | Powell, J., Atherton, H., Williams, V., Martin, A., Bennett, K., Bennett, A., … & Griffiths, K. M. (2017). Effectiveness and cost-effectiveness of a fully self-guided internet-based intervention for sub-clinical social anxiety symptoms: Protocol for a | Powell, J., Williams, V., Atherton, H., Bennett, K., Yang, Y., Davoudianfar, M., … & Griffiths, K. M. (2020). Effectiveness and cost-effectiveness of a self-guided internet intervention for social anxiety symptoms in a general population sample: |

| | | | |
|---|---|---|---|
| | | randomised controlled trial. *Digital health*, *3*, 2055207617702272. | randomized controlled trial. *Journal of medical Internet research*, *22*(1), e16804. |
| 34 | ISRCTN64826171 | Todd, N. J., Solis-Trapala, I., Jones, S. H., & Lobban, F. A. (2012). An online randomised controlled trial to assess the feasibility, acceptability and potential effectiveness of 'Living with Bipolar': a web-based self-management intervention for bipolar disorder: trial design and protocol. *Contemporary Clinical Trials*, *33*(4), 679-688. | Todd, N. J., Jones, S. H., Hart, A., & Lobban, F. A. (2014). A web-based self-management intervention for bipolar disorder 'living with bipolar': a feasibility randomised controlled trial. *Journal of affective disorders*, *169*, 21-29. |
| 35 | ISRCTN17308399 | Freeman, D., Yu, L. M., Kabir, T., Martin, J., Craven, M., Leal, J., … & Waite, F. (2019). Automated virtual reality (VR) cognitive therapy for patients with psychosis: study protocol for a single-blind parallel group randomised controlled trial (gameChange). *BMJ open*, *9*(8), e031606. | Freeman, D., Lambe, S., Kabir, T., Petit, A., Rosebrock, L., Yu, L. M., … & West, J. (2022). Automated virtual reality therapy to treat agoraphobic avoidance and distress in patients with psychosis (gameChange): a multicentre, parallel-group, single-blind, randomised, controlled trial in |

| | | | |
|---|---|---|---|
| | | | England with mediation and moderation analyses. *The Lancet Psychiatry*, *9*(5), 375-388. |
| 36 | ISRCTN12890709 | Williams, C., McClay, C. A., Martinez, R., Morrison, J., Haig, C., Jones, R., & Farrand, P. (2016). Online CBT life skills programme for low mood and anxiety: study protocol for a pilot randomized controlled trial. *Trials*, *17*(1), 1-7. | Williams, C., McClay, C. A., Martinez, R., Morrison, J., Haig, C., Jones, R., & Farrand, P. (2022). Online Cognitive Behavioral Therapy (CBT) Life Skills Program for Depression: Pilot Randomized Controlled Trial. *JMIR formative research*, *6*(2), e30489. |
| 37 | ISRCTN73535163 | Gellatly, J., Bower, P., McMillan, D., Roberts, C., Byford, S., Bee, P., ... & Lovell, K. (2014). Obsessive Compulsive Treatment Efficacy Trial (OCTET) comparing the clinical and cost effectiveness of self-managed therapies: study protocol for a randomised controlled trial. *Trials*, *15*(1), 1-10. | Lovell, K., Bower, P., Gellatly, J., Byford, S., Bee, P., McMillan, D., ... & Roberts, C. (2017). Low-intensity cognitive-behaviour therapy interventions for obsessive-compulsive disorder compared to waiting list for therapist-led cognitive-behaviour therapy: 3-arm randomised controlled trial of clinical effectiveness. *PLoS medicine*, *14*(6), e1002337. |

| | | | |
|---|---|---|---|
| 38 | ISRCTN34966555 | Bucci, S., Barrowclough, C., Ainsworth, J., Morris, R., Berry, K., Machin, M., ... & Haddock, G. (2015). Using mobile technology to deliver a cognitive behaviour therapy-informed intervention in early psychosis (Actissist): study protocol for a randomised controlled trial. *Trials*, *16*(1), 1-10. | Bucci, S., Barrowclough, C., Ainsworth, J., Machin, M., Morris, R., Berry, K., ... & Haddock, G. (2018). Actissist: proof-of-concept trial of a theory-driven digital intervention for psychosis. *Schizophrenia bulletin*, *44*(5), 1070-1080. |
