# Supplementary material for: Identifying and Categorizing Adverse Events in Trials of Digital Mental Health Interventions: Narrative Scoping Review of Trials in the International Standard Randomized Controlled Trial Number Registry
Source: JMIR Ment Health. 2023 Feb 22;10:e42501. doi: 10.2196/42501 (PMC9996423; doi:10.2196/42501)
Supplement: Multimedia Appendix 3 [file mental_v10i1e42501_app3.pdf]

Multimedia Appendix 3: Trial characteristics

	ISRCTN	Publication Year (protocol, final results)	Country	Method of Delivery	Comparators	Ages	Condition	Type of Intervention
16	ISRCTN12929657	2016, 2018	Netherlands	VR[a]	w/l[b] + TAU[c]	18+	Diagnosis of a psychotic disorder	VR exposure therapy
17	ISRCTN81375447	2009, 2011	UK	Web/ internet	TAU	18+	Diagnosis of bipolar disorder and current clinical remission	Internet psychoeducational intervention; Beating Bipolar
18	ISRCTN03704676	2014, 2015	Ireland	Web/ internet	w/l	18+	Mild to moderate depressive symptoms	Internet Cognitive Behavioural Therapy (iCBT)
19	ISRCTN24874457	2008, 2011	Netherlands	Web/ internet	w/l	18+	Diagnosis of diabetes, mild to severe depressive symptoms	iCBT
20	ISRCTN65657330	2010, 2012	Australia	Web/ internet	Purpose built information website	18+	Mild to severe psychological distress	Internet support group and training programme

21	ISRCTN40484777	2012, 2017	Germany/ Switzerland	Web/ internet	Face-to-face CBT	18+	Diagnosis of binge eating disorder	Internet-based self help
22	ISRCTN16303842	2014, 2016	Ireland	Web/ internet	w/l	18+	Diagnosis generalised anxiety disorder, mild to moderate	iCBT
23	ISRCTN31219579	2014, 2017	UK	Computer	TAU	12-18 yrs	Mild to moderate low mood or depression	Computerised CBT (cCBT)
24	ISRCTN82388279	2015, 2020	Spain	Web/ internet	Purpose built psychoeducation healthy lifestyle programme	18+	Diagnosis of Major Depression or Dysthymia, mild to moderate	iCBT + face to face care
25	ISRCTN25824611	2016, 2019	Malaysia	VR	Information pamphlet	18+	Phobia checklist	VR exposure therapy
26	ISRCTN12673428	2017, 2021	UK	Web/ internet	w/l + information website	16+	Mild to moderate depression, moderate to severe anxiety	Internet support and self-help; Big White Wall
27	ISRCTN32448671	2017, 2021	UK	Smartphone app	TAU	18+	Diagnosis of schizophrenia spectrum psychosis	App + face to face CBT
28	ISRCTN91967124	2018, 2020	UK	Web/ internet	w/l	18+	Moderate to severe depression and/or anxiety	iCBT

29	ISRCTN12765810	2020, 2020	UK/ USA	Smartphone app	w/l	18+	Moderate to severe anxiety	iCBT
30	ISRCTN70758207	2019, 2021	UK	Web/ internet	Psychoeducation website	9-17 yrs	Diagnosis of tic disorder	Internet behavioural therapy
31	ISRCTN10004994	2019, 2020	UK	Smartphone app	TAU	16+	Diagnosis of psychosis	Self-help app
32	ISRCTN14818949	2019, 2021	UK	Computer	TAU	4-7 yrs	Social Communication Behaviour Checklist	Computer game
33	ISRCTN15819951	2017, 2020	UK	Web/internet	w/l	18+	Score above 13 on Social Phobia Inventory	iCBT
34	ISRCTN64826171	2012, 2014	UK	Web/internet	TAU	18-65 yrs	Self-reported diagnosis of Bipolar Disorder Type I or II and above caseness on Mood Disorders Questionnaire	Internet self-help
35	ISRCTN17308399	2019, 2022	UK	VR	TAU	16+	Clinical Diagnosis of ICD-10 codes F20-29 or F31.2, 31.5, 32.3, 33.3	VR cognitive therapy
36	ISRCTN12890709	2016, 2022	UK	Web/internet	w/l	18+	Score over 10 on PHQ-9	iCBT

37	ISRCTN73 535163	2014, 2017	UK	Web/internet	w/l, guided self-help	18+	Met DSM-IV criteria for OCD (assessed using the Mini-International Neuropsychiatric Interview and scoring 16+ on the Yale-Brown Obsessive Compulsive Checklist–Self-Report (Y-BOCS-SR)	cCBT
38	ISRCTN34 966555	2015, 2018	UK	Smartphone app	TAU	16+	In current contact with an early intervention for psychosis service	CBT app

[a] virtual reality, [b] waitlist control, [c] treatment as usual
