# Supplementary material for: Identifying and Categorizing Adverse Events in Trials of Digital Mental Health Interventions: Narrative Scoping Review of Trials in the International Standard Randomized Controlled Trial Number Registry
Source: JMIR Ment Health. 2023 Feb 22;10:e42501. doi: 10.2196/42501 (PMC9996423; doi:10.2196/42501)
Supplement: Multimedia Appendix 4 [file mental_v10i1e42501_app4.pdf]

Multimedia Appendix 4: Adverse event reporting in trials

	ISRCTN	References to AEs	Reporting of AEs	Definitions of AEs	Seriousness of AEs	Expectedness	Monitoring of AEs	Relatedness of AEs
16	ISRCTN 12929657	Protocol, published results publication (PRP)	No AEs	None provided				
20	ISRCTN 65657330	CONSORT checklist	AEs not applicable	None provided				
21	ISRCTN 40484777	Protocol, PRP	AEs and SAEs	Fatal or life-threatening events, dropouts or withdrawals	SAEs were fatal or life-threatening		AEs reported to principal investigator, ethics committee, and data monitoring ethics committee.	

23	ISRCTN 31219579	Protocol		Short Mood and Feelings Questionnaire, fatal or life-threatening, unexpected adverse reactions	SAEs were fatal or life-threatening	Stated they were monitoring only those related to the nature of the condition, implying expectedness	SAEs reported to research ethics committee, sessional collection of data, unexpected AEs reported to Data Monitoring Ethics Committee (DMEC), Trial Steering Committee (TSC), trial sponsor and ethics committee	
24	ISRCTN 82388279	CONSORT checklist	No AEs	None provided				

26	ISRCTN 12673428	CONSORT checklist, protocol	No AEs	None provided				
27	ISRCTN 32448671	Protocol, PRP	AEs and SAEs	AEs include: deaths; self-harm; serious violent incidents; complaints about therapy; and referrals to crisis care or admission to psychiatric hospital during therapy.	A standard method of reporting will be employed, categorising events by severity (five grades, A–E).	Indicated investigators would determine if an AE was expected or unexplained in protocol but did not report in PRP	Monitored using SPIRIT guidance. Subject to the approval by the independent chairperson of the Data Monitoring and Ethics Committee (DMEC, see below, 'Research governance'), investigators will also determine	Protocol: The event was rated within five categories from 'not related' to 'related'. Researchers determined whether an event was temporally related to the intervention, unexpected or unexplained based on participant's clinical course, previous conditions

							whether an event is temporally related to the intervention, and whether it is unexpected or unexplained given the participant’s clinical course, previous conditions and history, and concomitant treatments.	and history, and concomitant treatments
28	ISRCTN 91967124	Protocol, PRP	AEs	None provided in protocol. Identified as deterioration within PRP				

29	ISRCTN 12765810	Protocol, PRP	AEs	The study will monitor for potential adverse effects through frequent Anxiety and Depression symptom questionnaires (GAD-7 and PHQ-9). A modified symptom checklist will also be used to document potential somatic and psychological side effects. The questionnaire is administered at post-intervention only and asks participants to indicate whether or not they experienced any of 14 pre-specified unwanted symptoms (e.g. low mood, feeling agitated) or other	A serious adverse event is defined as any untoward medical occurrence that is believed by the investigators to be causally related to digital CBT and results in any of the following: life-threatening condition (that is, immediate risk of death); or severe or permanent disability, prolonged hospitalisation, or a significant hazard		Trial management committee monitored SAEs	Protocol: An SAE is any event considered to be causally related to digital CBT by the investigators

				non-listed unwanted symptoms at any point during the treatment period.	as determined by the trial management committee.			
30	ISRCTN 70758207	Protocol, PRP, ISRCTN	AEs and SAEs	Adverse events/side effects will be recorded on a modified version of the side<br>effects scale developed by Hill and Taylor.	Appendix: Seriousness is assessed in the first instance by the trial manager and the researchers who reported the AE, if there are any concerns or doubts with seriousness or relation to the intervention the Chief Investigator (CI) is informed. The CI is informed	Reported both expected and unexpected in final report	All adverse events will be recorded and monitored, and the chief investigator and medical expert (CH) will determine seriousness and causality and report the event to the DMEC and ethics committee where	Protocol: the chief investigator will determine causality.<br>PRP: an independent committee deemed SAEs either related or unrelated

					of all SAEs and is responsibility for making final categorisation. The protocol defines an SAE as any untoward occurrence that:<br>• Results in death,<br>• Is life-threatening,<br>• Requires hospitalisation or prolongation of existing hospitalisation,<br>• Results in persistent or significant		necessary. Side effects will be formally monitored via completion of the side-effects scale throughout the intervention and for 3 months after the intervention finishes (month 6).<br>Publication: Participants were also encouraged to report adverse effects to their	

					disability or incapacity, or<br>• Consists of a congenital anomaly or birth defect<br>• Is otherwise considered medically significant by the investigator		therapist or outcome assessor.	
31	ISRCTN 10004994	Protocol, PRP, CONSORT checklist	No AEs	Serious adverse events such as hospital admissions and death reported to the trial team will be reviewed by the chief investigator	Serious adverse events such as hospital admissions and death		Serious adverse events such as hospital admissions and death reported to the trial team will be reviewed by the chief investigator	Protocol: Identified adverse events assessed as trial-related will be reported to the trial sponsor.<br>PRP: no research-related SAEs were recorded

							Identified adverse events assessed as trial-related will be reported to the trial sponsor.	
32	ISRCTN 14818949	PRP	No AEs	None provided				
33	ISRCTN 15819951	Protocol, PRP	No AEs	None provided		Did not expect AEs		
35	ISRCTN 17308399	Protocol, PRP	AEs and SAEs	An adverse event is defined by the ISO14155:2011 guidelines for medical device trials as serious if it: (a) results in death or, (b) is a life-threat- ening illness or injury or, (c) requires hospitalisation or prolongation of existing			AEs monitored by self-report and reviewing of medical notes at the end of the trial. Reviewed by DMEC	PRP: an independent committee assessed relatedness of SAEs with a rating from definitely not to definitely related.

				hospitalisation or, (d) results in persistent or significant disability or incapacity or, (e) medical or surgical intervention is required to prevent any of the above, (f) leads to foetal distress, foetal death or consists of a congenital anomaly or birth defect or (g) is otherwise considered medically significant by the investigator.				
37	ISRCTN 73535163	CONSORT checklist	AEs not applicable	None provided				
38	ISRCTN 34966555	Protocol, PRP	No SAEs	None provided			Serious adverse events are regularly	

							monitored and documented by the research team and reported immediately to the chief investigator and/or a senior clinical member of the team. Any identified adverse event is then discussed with a nominated senior clinical academic independent to the University of	

							Manchester and the research team and an appropriate course of action is agreed and implemented.	
