# Supplementary material for: Identifying and Categorizing Adverse Events in Trials of Digital Mental Health Interventions: Narrative Scoping Review of Trials in the International Standard Randomized Controlled Trial Number Registry
Source: JMIR Ment Health. 2023 Feb 22;10:e42501. doi: 10.2196/42501 (PMC9996423; doi:10.2196/42501)
Supplement: Multimedia Appendix 5 [file mental_v10i1e42501_app5.pdf]

Multimedia Appendix 5: Potential adverse events identified in trials that made no reference to AEs or reported no AEs (n=16).

	ISRCTN	AEs	Support and Training	Dropouts	Strategies to manage risk	Inclusion Criteria	Exclusion Criteria
16	ISRCTN 12929657	No AEs	Direct*: therapist remains in control of the VR. They receive training and are rated for fidelity.	Death, sickness, fear of VR, substance abuse,	..	To be eligible to participate, patients must meet all of the following criteria: DSM IV diagnosis of a psychotic disorder according to the Mini International Neuropsychiatric Interview (MINI), Avoiding either shops, streets, public transportation or bars/restaurants as assessed by the Safety Behavior Questionnaire (SBQ), A paranoia score (>40) as assessed by the Green et al.	IQ ≤70. IQ must be established by a valid instrument, such as the Wechsler Adult Intelligence Scale or Wechsler Intelligence Scale for Children. Information on IQ can be found in the status chart of the patient. In case of doubt, the short form of the WAIS III is used to assess IQ, Insufficient command of the Dutch language, Epilepsy. If no epilepsy is mentioned in the patient status, this is checked with the patient

						Paranoid Thoughts Scale (GPTS), Age 18–65 years	
17	ISRCTN 81375447	Not referenced	Brief†: initial meeting with consultant psychologist to introduce programme. Forum moderated by consultant psychologist. No training specified.	Not reported	..	The main inclusion criteria for this study were age between 18 and 65 and a diagnosis of DSM-IV bipolar disorder [including type I and type II/not otherwise specified (NOS)] currently in clinical remission. Diagnosis was confirmed using the Mini International Neuropsychiatric Interview (MINI) (11) and clinical remission was defined as not fulfilling diagnostic criteria for a depressive, manic or mixed affective episode	The exclusion criterion was an inability to engage fully in the psychoeducational programme, for example, because of cognitive impairment or not having English language ability of sufficient level. Given that this was an exploratory trial, no other exclusion criteria were specified.

						during the preceding three-month period, plus a current Montgomery–Åsberg Depression Rating Scale (MADRS) (12) score of ≤ 10 and a Young Mania Rating Scale (YMRS) (13) score of ≤ 8. These MADRS and YMRS threshold scores are widely accepted correlates of symptomatic remission in bipolar disorder.	
18	ISRCTN 0370467 6	Not referenced	Remote‡: trained and supervised supporters offered feedback using a dashboard where they can see participants' progress.	Not reported	Reviewed all communication with participants	18 years of age, Mild to Moderate depressive symptoms (BDI-II 14–29)	Suicidal intent/ideation: score >2 on BDI-II question 9, Psychotic illness, Currently in psychological treatment for depression, On medication for less than 1 month, Alcohol or drug misuse, Previous

							diagnosis of an organic mental health disorder, Depression preceding or coinciding a diagnosed medical condition
19	ISRCTN 24874457	Not referenced	Remote: coaches (psychologists supervised by research team) offered feedback and encouragement by email.	Not reported	..		A history of suicide attempt(s) or current suicidal ideation; bipolar depression or psychotic disorder; pregnancy; recent loss of a significant other (<6 months ago); and insufficient Internet literacy
20	ISRCTN 65657330	AEs not applicable	Remote: the Board was moderated by a member of the research team. Telephone call reminders from research team.	Lack of access, no benefit, too busy, change in circumstances	Prevented participants discussing certain topics (e.g. suicide)	Age 18 to 65, Kessler Psychological Distress score >22, home or work access to the internet, consent to participate	Self-reported current or imminent treatment from a mental health professional or mutual support group at the time of recruitment, current treatment with CBT, self reported current or past experience with or diagnosis of

							psychosis, schizo[phrenia or bipolar disorder; current or prior participation in another project conducted by the lead investigator's research centre
22	ISRCTN 1630384 2	Not referenced	Remote: trained supporters monitored progress and offered feedback asynchronously. All were psychology postgraduates.	Not reported	Asked participants to sign a user contract and a system for flagging those considered higher need	All registered students at the University of Dublin, Trinity College, Dublin, will be eligible to participate. The study will therefore consist of adult primary care patients fulfilling the Diagnostic and Statistical manual for Mental Health Disorders – Version 5 (DSM-5) [2] criteria for generalized anxiety symptoms. It is the case that all participants will have	Participants attending face-to-face counseling will be excluded.

						clinically meaningful generalized anxiety symptoms. Participants with comorbid disorders, such as mood disorders will be included once GAD is the primary diagnosis. On screening participants, eligibility criteria include that participants are at least 18 years of age and have a DSM-IV congruent score of 10 or above on GAD-7.	
23	ISRCTN 31219579	Not reported in final publication	Direct: researchers waited outside the room during sessions.	Sickness, change in circumstancs, dislike of programme, changing mind/withdrawal, unable to contact, refusal	..	Our target population will be adolescents aged 12–18 with low mood/depression. Our inclusion threshold will be a MFQ score of 20 or above, which has 70% sensitivity	We will exclude participants who are seeking to end their life, suffering psychotic symptoms or depressed in the postnatal period. Participants with previous depression or

						and 81% specificity for any mood disorder.19 The cut-off for a major depressive episode is 29. We will also include participants with either comorbid physical illness or comorbid non-psychotic functional disorders, such as anxiety.	previous treatment with antidepressants or experience of cognitive therapy will not be excluded. We will exclude cases of psychotic depression, since computerised therapy for this group is not recommended within NICE guidance.
24	ISRCTN 82388279	No AEs	Brief: groups were held at the beginning to introduce the programme after which automated messages were sent to participants every 2 weeks. No training specified although GPs	Not reported	Clinicians were asked to monitor patients	Older than 18 years, DSM-5 diagnose of Major Depression or Dysthymia, mild or moderate depression expressed as score lower than 14 in the Patient Health Questionnaire (PHQ) [29], depressive symptoms presented for at least two	Any diagnose of disease that may affect central nervous system (brain pathology, traumatic brain injury, dementia, etc.), any psychiatric disorder other than Major Depression, Dysthymia, anxiety disorders or personality disorders, any

			in improved TAU received training.			months, be able to read and understand Spanish language and enough capacity for understanding and signing the written informed consent form	medical, infectious or degenerative disease that may affect mood, presence of delusional ideas or hallucinations consistent or not with mood, and suicide risk.
25	ISRCTN 2582461 1	Not referenced	Direct: VR was operated by author.	Cybersickness	Panic button whilst in VR	Meeting the criteria of “Phobia checklist”., An age between 18 to 50 yr. Any dental phobic patient requiring the following planned dental treatment/s of at least 30 minutes per appointment. Restorative dental procedure which may or may not be requiring local anesthesia. Extraction procedure requiring local	Hearing or visual impairment such as stereoscopy blindness or nystagmus. Known mental disorders such as psychosis, post-traumatic stress disorder, developmental or intellectual disability and cognitive impairment. Known balance disorders such as vertigo and cybersickness. Patients with previous history of epileptic seizures. Any history of

						anesthesia.	cardiac problems. Patients who are undergoing, or have undergone, any cognitive behavioral therapy (CBT)-based intervention for dental phobia. Language impediment (cannot understand English). Patients wearing glasses of greater than plus 3.5 power.
26	ISRCTN 12673428	No AEs	Remote: trained counsellors helped facilitate interactions and moderate the forum.	Lack of personal interaction or clinical support, lack of technical support	..	Aged 16 years or over, Resident in the County of Nottinghamshire, Scores between 10 and 20 on the 9-item Personal Health Questionnaire (PHQ-9) [25] and/or 10 or more on the 7-item Generalized Anxiety Disorder (GAD-7) questionnaire [26],	Scores 21 or more on the 9-item Personal Health Questionnaire (PHQ-9, severe depression), Scores 2 or 3 on PHQ-9 item “thoughts that you would be better off dead or of hurting yourself in some way, Scores below 10 on PHQ-9 and 7-item Generalized Anxiety Disorder (GAD-7)

						indicating probable caseness for depression and anxiety, respectively, but not a definite diagnosis of depression or anxiety disorder, Access to the Internet through a computer, tablet, or smartphone (Windows, iPhone operating system [iOS, Apple Inc], and Android), Able and willing to give informed consent (through electronic consent)	questionnaire, BWW and Moodzone are only available in English. Therefore, the website will recommend to participants that if they do not feel that they are sufficiently proficient in the use of the English language, they need not take part. There will be no test of proficiency.
31	ISRCTN 10004994	No AEs	Direct: existing services were asked to provide support.	Lack of personal interaction or clinical support, concerns about data privacy, relapse, progressing to treatment	..	Aged 16 or older. Have experienced at least one episode of psychosis. Currently on the caseload of an EIP service and in contact with clinicians. User of a	Lack of capacity to provide informed consent to participate in the trial. Inability to communicate and understand English sufficiently to understand trial procedures

						Smartphone with an Android operating system"	and use My Journey 3. In the view of their EIP service, poses such a high risk to others that it would be unsafe to conduct research meetings even on NHS premises
32	ISRCTN 14818949	No AEs	Direct: brief support from trained teaching assistants.	Flood within the school	..	Suitable children using the Social Communication Behaviour Checklist. Four-to seven-years-old at the time of the SLT's assessment of eligibility. Have at least minimum levels of English (including any children with English as an additional language). Not suffering with a hearing, visual or physical	..

						impairment severely affecting speech production	
33	ISRCTN 1581995 1	No AEs	Remote:	Lack of access, change in circumstances, unable to contact		Initial inclusion criteria were having access to the internet-based intervention, aged 18 years or older, resident in England, having an email address and mobile telephone number (to receive study emails and text alerts), and an initial criterion of scoring in a subclinical range of 13 to 19 on the 17-item Social Phobia Inventory (SPIN-17). We had initially chosen the 13 to 19 range with expert advice as this would, in theory, capture those scoring above	We continued to exclude anyone receiving professional help, and therefore, the final sample represented adults in the general population who self-reported some level of social anxiety symptoms but who were not receiving treatment for social anxiety

						the population mean score (11-12) while excluding those scoring above the commonly used threshold of 19, which indicates further assessment may be warranted (although this threshold does not represent a diagnosis). However, early in recruitment, it became apparent that most people in the general population volunteering for this study scored much higher than this, and the distribution of SPIN-17 scores meant that very few scored in the low range. There was clear evidence of a high level of	

						unmet need among individuals living with social anxiety symptoms in the community and not seeking help elsewhere. With advice from our independent Trial Steering Committee, we, therefore, revised and reregistered the protocol (in line with good practice in clinical trials) to modify the inclusion criteria to include all individuals scoring 13 or more on SPIN-17, therefore capturing those in our hypothesized subclinical range of 13 to 19, as well as those with a higher score	

34	ISRCTN 64826171	Not referenced	Remote:	Too busy, change in circumstances, unable to contact	visible signposting within the intervention to support	Participants were eligible if they were aged 18–65, resident in the UK, with a self-reported clinical diagnosis of Bipolar Disorder Type I or II, and scoring above a threshold sensitive to diagnostic criteria for Bipolar Disorder Type I and II on the Mood Disorders Questionnaire (MDQ) (Hirschfeld et al., 2000; Twiss et al., 2008). For the purposes of the intervention, participants needed to understand written English, have access to a computer, the internet, an email account and a printer.	..

						All participants gave informed consent.	
36	ISRCTN 12890709	Not referenced	Remote: support is offered both through automated emails and telephone/emails once a week from support workers	Not reported	..	Aged 18 or over. Living in the UK. Ability to understand written and spoken English language. Regular access to a computer, smart phone or tablet with audio and broadband connection. Score of 10 or more on the Patient Health Questionnaire	High rating of suicidality (i.e., scoring 2 or 3 on item 9 of the Patient Health Questionnaire 9). Currently receiving any psychological intervention, such as counselling or psychotherapy. New or altered dose of antidepressant in the past month. Taking part in any other research projects
37	ISRCTN 73535163	AEs not applicable	Remote: six 10 minute phone calls for risk assessment, progress review and problem solving from a	Not reported	…	(1) aged 18+ years, (2) able to read English, (3) currently waiting for access to therapist-led CBT, (4) meeting DSM-IV criteria for OCD (assessed using the	(1) experiencing active suicidal or psychotic thoughts, (2) meeting DSM-IV alcohol or substance dependence criteria, (3) receiving psychological treatment for

			psychological wellbeing practitioner			Mini-International Neuropsychiatric Interview), and (5) scoring 16+ on the Yale-Brown Obsessive Compulsive Checklist–Self-Report (Y-BOCS-SR).	OCD, or (4) with language difficulties that would preclude participation.
38	ISRCTN34 966555	No SAEs	Brief: participants received a training session via phone on how to set up and use app	Only one participant withdrew in a non-research related incident		(1) in current contact with an Early Intervention Service in the North West of England; (2) capacity to provide informed consent; and (3) English language proficient.	(1) aged less than 16 years at point of recruitment; (2) not capable of giving informed consent; (3) non-English proficient; and (4) inpatient at point of recruitment.
