# Supplementary material for: Identifying and Categorizing Adverse Events in Trials of Digital Mental Health Interventions: Narrative Scoping Review of Trials in the International Standard Randomized Controlled Trial Number Registry
Source: JMIR Ment Health. 2023 Feb 22;10:e42501. doi: 10.2196/42501 (PMC9996423; doi:10.2196/42501)
Supplement: Multimedia Appendix 6 [file mental_v10i1e42501_app6.pdf]

Multimedia Appendix 6: Potential adverse events identified in trials that reported AEs and SAEs (n=6).

	ISRCTN	AEs	Support and Training	Dropouts	Strategies to manage risk	Inclusion Criteria	Exclusion Criteria
21	ISRCTN 40484777	AEs and SAEs	Brief: coaches in guided self-help arm provided support and encouragement asynchronously. They also met at the start and end of the programme.		..	Diagnostic criteria for BED according to DSM-IV, or Subsyndromal BED: Patients have to meet the criteria for OBEs but can lack one of the other DSM-IV criteria (frequency of less than 2 days with OBEs in 6 months, no marked distress, or presence of only 2 instead of 3 of the 5 associated criteria), Age 18 years or older, 27 ≤ BMI < 40 kg/m2, Written informed consent of the patient	Current bulimia nervosa, Current substance abuse, Current suicidal ideation, Psychotic disorder, Bipolar disorder, Serious unstable medical problems or conditions (for example, type 1 diabetes mellitus or thyroid problems) that influence weight or eating, Ongoing psychotherapy, Current intake of antipsychotic or weight-affecting drugs, Pregnancy or lactation

27	ISRCTN 32448671	AEs and SAEs	Direct: trained therapists delivered face-to-face component.	Not reported	..	18 years or older; persistent (≥3 months) distressing paranoia (assessed using the Schedules for Clinical Assessment in Neuropsychiatry23); score of greater than 29 on the Green et al Paranoid Thoughts Scale (GPTS) Part B, the Persecutory subscale24; a diagnosis of schizophrenia spectrum psychosis (codes F20-29 from the International Statistical Classification of Diseases and Related Health Problems, Tenth Revision)25; capacity to provide informed consent; and sufficient English to participate in trial processes	Participants were excluded if they had profound visual or hearing impairment, were unable to engage in assessments, were currently receiving psychological therapy for paranoia, and had a primary diagnosis of substance use disorder, personality disorder, organic syndrome, or learning disability.

28	ISRCTN 91967124	AEs	Remote: trained and supervised PWP provided asynchronous feedback via the programme.	Withdrawal, step up in treatment, trial confusion, unable to contact, increased risk, drug use, errors, referred to secondary care, identified risk, received treatment	..	Minimum age of 18 years, a score of greater than or equal to 9 on PHQ9 and/or of 8 on GAD7, suitable for an internet-delivered intervention	Suicidal intent or ideation: score > 2 on PHQ9 question 9, psychotic illness, currently in psychological treatment for depression and/or anxiety, alcohol or drug misuse, previous diagnosis of an organic mental health disorder
29	ISRCTN 12765810	AEs	None specified.	Not reported	..	1) be aged 18 years or older; 2) score 10 or higher on the GAD-7 [35] indicating at least moderate GAD symptom severity; 3) screen positive for a GAD diagnosis on a digital	1) being diagnosed with any of the following conditions: schizophrenia, psychosis, bipo-lar disorder, seizure disorder, substance use disorder; 2) having recent trauma to the head

						ver- sion of the Mini-International Neuropsychiatric Inter- view (MINI) version 7 for DSM-5 , followed by telephone verification; 4) be either not on prescription medication for anxiety, depressive symptoms, or poor sleep, or on a stable dose for at least 4 weeks; and 5) must not be currently receiv- ing or have previously received CBT for anxiety in the last 12 months (this inclusion criterion was included after a modification was made by the ethical review committee after recruitment start).	or brain damage; 3) having severe cognitive impairment; 4) having serious physical health concerns necessitating surgery or with a prognosis of less than 6 months; or 5) being pregnant. Individuals with other anxiety or related disorders (e.g. panic disorder, social anxiety disorder, and so forth) were included in the study as long as they endorsed worry (consistent with a GAD diagnosis) as their primary concern assessed at telephone screening.

30	ISRCTN 70758207	AEs and SAEs	Remote: trained and supervised researchers provided asynchronous feedback to motivate and support.	Not reported	..	Aged 9–17 years. Suspected or confirmed TS or chronic tic disorder. – Including moderate/severe tics: Total Tic Severity Score >15 on the YGTSS; TTSS score >10 if motor or vocal tics only. Competent to provide written, informed consent (pa- rental consent for child aged <16 years). Broadband internet access and regular PC/laptop/ Mac user, with mobile phone SMS.	Receipt of/engaged in structured behavioural intervention for tics (eg, HRT/CBIT or ERP) within the last 12 months. Change to medication for tics (start or stop) within the previous two months. Diagnoses of alcohol/substance dependence, psycho- sis, suicidality or anorexia nervosa. Moderate/severe intellectual disability. Immediate risk to self or others. Parent or child not able to speak or read/write English.
35	ISRCTN 17308399	AEs and SAEs	Direct: VR delivered by trained mental health workers	Not reported	..	Eligible patients were adults aged 16 years or older, who were attending an NHS mental health trust for the treatment	An inability to attempt an Oxford-Behavioural Assessment Task (O-BAT) at baseline for practical reasons (eg, due to not

						of psychosis, with a clinical diagnosis of schizophrenia spectrum psychosis (ICD-10 codes F20–29) or an affective diagnosis with psychotic symptoms (F31.2, 31.5, 32.3, 33.3),had self-reported difficulties going outside the home primarily due to anxiety (for which they would like to have treatment), and were willing and able to provide informed consent for participation in the trial.	being permitted to leave a psychiatric ward); photosensitive epilepsy; substantial visual, auditory, or balance impairment; current receipt of another intensive psychological therapy; insufficient comprehension of English; currently in a forensic setting or Psychiatric Intensive Care Unit; organic syndrome; primary diagnosis of alcohol or substance use disorder or personality disorder; clinically significant learning disability; or current active suicidal intent with plans (ie, a crisis point). Written informed consent was obtained before participation
